# Regional Differences and Trends Within Texas in HPV Vaccination Among Medicaid-insured Adolescents

**DOI:** 10.1177/21501319261432414

**Published:** 2026-03-27

**Authors:** Erika L. Thompson, Yong Shan, Kirsten Y. Eom, Miranda E. Cano, Yong-Fang Kuo

**Affiliations:** 1 Kate Marmion School of Public Health, University of Texas at San Antonio, San Antonio, TX, USA; 2 University of Texas Medical Branch, Galveston, TX, USA

**Keywords:** HPV vaccination, Medicaid, adolescent, Texas

## Abstract

**Objectives:**

The purpose of this study was to compare regional HPV vaccination rates and trends among the Texas Medicaid population of adolescents in 2019 to 2021.

**Methods:**

We constructed 3 annual cross-sectional cohorts from Texas Medicaid data from 2019 to 2021, including a total of 1 531 301 individuals contributing 2 757 577 person-years of adolescents aged 9-18 for this analysis. The primary outcome was an observed HPV vaccine dose during the study period. We conducted multivariable logistic regression to estimate the odds of HPV vaccination based on region and the socio-demographic predictor variables.

**Results:**

Overall, the HPV vaccination incidence rate was 13.1% for Medicaid adolescent clients in Texas. The 2020 and 2021 cohorts were significantly less likely to be vaccinated for HPV than the 2019 cohort. Regional variation was detected for adolescents. Significant differences were observed in both age cohorts by sex, race/ethnicity, urbanicity, and cohort year.

**Conclusion:**

Declines in HPV vaccination after the COVID-19 pandemic among Texas Medicaid-insured adolescents are of urgent concern if HPV-related cancers are to be prevented. Furthermore, regional differences are apparent throughout Texas, which may contribute to an unequal burden of future disease.

## Introduction

Human papillomavirus (HPV) is responsible for 39 300 cancers per year in the United States, including cervical, anal, oropharyngeal, vaginal, vulval, and penile cancers.^1^ These cancers are largely preventable (>90%) through HPV vaccination—a safe and effective vaccine.^2^ Adolescents ages 11 to 12 are recommended to receive this routine vaccination series and can start the series as early as age 9. Those not vaccinated by age 12 are eligible for catch-up HPV vaccination until age 26 in the United States.^3^ Despite the availability of this primary prevention tool, rates of uptake are lower than those of other adolescent-recommended vaccines. As of 2024, 62.9% of adolescents ages 13 to 17 were up to date on their HPV vaccination series compared to 92.2% for Tdap and 90.1% for meningococcal.^4^ Moreover, the HPV vaccination rates have largely stagnated since the COVID-19 pandemic,^4^ which resulted in major interruptions to primary care in 2021.

One out of 10 adolescents in the United States lives in Texas.^5^ Yet, Texas continues to be one of the states with the lowest HPV vaccination rates nationally. As of 2024, 52.2% of adolescents ages 13-17 were up to date on their HPV vaccination series in Texas.^6^ HPV vaccination rates vary across Texas, yet this regional variation is not consistently documented, resulting in a significant data gap.^6^ Further compounding the data gap is that the Texas immunization registry is opt-in, so it does not include all information on youth vaccination.^7^ This issue is especially important for children in the Medicaid program, who comprise an estimated 50.4% of all children in the state.^8^ While this group generally has higher HPV vaccination rates due to federal programs that remove cost barriers,^6^ this statewide success may obscure regional disparities. It is therefore essential to investigate why and where regional disparities in vaccination uptake persist within the Medicaid population, as these children continue to face multifaceted, location-based barriers to care. Furthermore, routine data on young adult HPV vaccination is nonexistent, which limits our ability to do routine surveillance of HPV vaccination among this catch-up population. While Medicaid claims data cannot estimate the prevalence of HPV vaccination among this dynamic population, claims data can describe differences among groups and trends over time based on observed HPV vaccination doses during the study period.

Understanding the problem at a local level is a significant challenge. A substantial data gap exists because routine reporting does not capture regional disparities, especially in medically underserved areas. These are often the same areas with high concentrations of children on Medicaid whose vaccination rates—boosted by state and federal programs—can mask the local disparities and barriers. Additionally, the Cancer Prevention and Research Institute of Texas (CPRIT) has invested heavily in these communities since 2010,^9^ which may improve HPV vaccination rates in these regions. The purpose of this study was to compare regional HPV vaccination rates over time among the Texas Medicaid population of adolescents in 2019-2021. An exploratory aim was to determine if regions with more CPRIT-funded prevention projects had higher HPV vaccination rates among the Texas Medicaid population.

## Methods

### Data

We used Texas Medicaid administrative data, including member, inpatient and outpatient claims and encounters, from 2018 to 2021. We linked Medicaid administrative data with *the American Community Survey by resident zip code, and* Rural-Urban Continuum Codes by resident county code.

### Cohort

We constructed 3 annual cross-sectional cohorts from Texas Medicaid data from 2019 to 2021, including 7 643 768 individuals contributing to 18,040,161 person-years (individuals could contribute more than one person-year). For each annual cohort, we selected children and adults eligible for routine HPV vaccination, ages 9-18, and excluded anyone with a documented pregnancy/delivery in the year of analysis or HPV vaccination in the prior year. Furthermore, to be included in the analytic sample, participants needed to be continuously enrolled for a 2-year period (current and prior year) in Texas Medicaid. Finally, participants with missing data based on sex or location were excluded. Combining 3 annual cohorts resulted in a final sample size of 1,531,301 participants contributing to 2 757 577person-years. The University of Texas Medical Branch Institutional Review Board reviewed and exempted this study from full review.

### Variables

The primary outcome measures a binary indication of HPV vaccination initiation (yes/no). ICD-10 codes (90649 and 90651) were used to identify HPV vaccine outpatient claims or other services. Individual-level sociodemographic characteristics included sex (male, female), race/ethnicity (non-Hispanic White, non-Hispanic Black, non-Hispanic Asian, Hispanic, and Other/Missing). The year of data was also included—2019, 2020, and 2021.

We measured 4 community-level variables. Urbanicity of the county of residence (metropolitan, urban, and rural) was based on 2013 Rural-Urban Continuum Codes. We measured the educational attainment level of the zip code of residence at the time of vaccination, which was operationalized as the percent that had received a high school degree or GED (<69.9%, 70.0%–80.8%, 80.9-88.2%, and >88.2%) according to the American Community Survey 5-Year Estimates from 2020. To account for regional differences within Texas, a person’s county of residence was categorized into the 11 Public Health Regions for the state.^10^ Texas has 254 counties and is divided into these public health regions to support public health work across counties. Finally, CPRIT Prevention Program Administrators collect data on the number of HPV-related CPRIT Prevention Projects in each county each year. Using data from the CPRIT Program, for each year, we measured the regional sum of HPV-related CPRIT-funded prevention projects in the counties within each respective region divided by the number of counties in a public health region, which is presented as a ratio.

### Data Analysis

All analyses were performed using SAS Enterprise version 9.4.^11^ We conducted descriptive statistics overall and by the outcome variable for the full sample. Chi-square tests and *t*-tests were used to test differences in predictor variables by HPV vaccination status. Next, we conducted multivariable logistic regression to estimate the odds of HPV vaccination based on the predictor variables. We calculated the incidence rate of HPV vaccination by public health region and year to examine trends in vaccination initiation. Subsequently, we examined the association of the number of CPRIT-funded prevention projects in a public health region in prior years (2019–2020) to HPV vaccination initiation in 2020-2021. The lag time accounted for project start-up for the impact on initiation rates. The region was removed from the model due to multicollinearity with the number of projects by region. Odds ratios and 95% confidence intervals are reported.

## Results

### Description of the Cohort

There was a relatively even split in sex, and most enrollees were Hispanic and lived in metropolitan areas (Table 1). Approximately half of the enrollees lived in zip codes where 80% or more had a high school degree. The Texas Public Health Regions with the most representation in the cohort were Regions 3 and 6. On average, there were 2.4 CPRIT-funded projects per county in each region. The cohort year with the most person-years was 2021. The HPV vaccination incidence rate was 13.1% for 9-18 year olds. There were statistically significant differences by HPV vaccination for individual and community-level demographics, including region.

**Table 1.** Baseline Characteristics of Texas Medicaid Enrollees Aged 9 to 18 Years by HPV Vaccination Initiation, 2019 to 2021.

Total person-years, row (%)	Total	Unvaccinated	Vaccinated
	2 757 577 (100.0)	2 397 276 (86.9)	360 301 (13.1)
Cohort year*, column (%)			
2019	26.9	26.4	30.1
2020	29.9	30.2	27.6
2021	43.2	43.4	42.3
Demographic			
Sex*			
Female	48.1	48.0	48.9
Male	51.9	52.0	51.1
Race/ethnicity*			
Non-Hispanic White	14.6	15.1	11.3
Non-Hispanic Black	15.1	15.3	14.1
Non-Hispanic Asian	1.4	1.4	1.4
Hispanic	59.7	58.8	65.4
Other/missing	9.2	9.4	7.8
Community-level			
Urbanicity of residence*			
Metro	87.8	87.4	89.8
Rural	0.8	0.9	0.6
Urban	11.4	11.7	9.6
Percent of high school degree per zip code*			
<70.0	26.1	25.7	28.9
70.0–80.8	25.9	25.8	26.6
80.9–88.2	24.3	24.4	23.3
>88.2	23.7	24.1	21.3
Texas Public Health Region*			
1 – Northwest (Lubbock)	3.1	3.2	2.6
2 – North (Abilene)	1.9	2.0	1.5
3 – North (Arlington)	22.9	23.1	21.5
4 – Northeast (Tyler)	4.4	4.5	3.7
5 – East Texas	3.1	3.2	2.5
6 – Southeast (Houston)	24.1	23.8	26.3
7 – Central (Austin)	9.1	9.2	8.8
8 – South Central (San Antonio)	11.0	11.0	11.0
9 – West (Odessa)	1.8	1.9	1.4
10 – West Border (El Paso)	4.1	4.0	4.5
11 – South Border (Harlingen)	14.5	14.3	16.0
Mean Ratio of CPRIT Projects per County (SD)*	2.43 (1.38)	2.41 (1.37)	2.53 (1.39)

Abbreviation: SD, standard deviation.

Urbanicity of residence is based on RUCC. Ratio of CPRIT projects per county was measured as the regional sum of HPV-related CPRIT-funded prevention projects in the counties within each respective region divided by the number of counties in a public health region.

* *P*-value < .05.

Table 2 presents the stratified multivariable models estimating the odds of HPV vaccination initiation. All Texas Public Health Regions were significantly more likely to have higher incidence rates of HPV vaccination compared to Region 1 (Northwest), except for Region 9 (West). Region 9 had significantly lower relative HPV vaccination incidence rates compared to all other regions. Furthermore, Region 7 (Austin), Region 10 (El Paso), and Region 11 (Harlingen) had significantly higher relative HPV vaccination incidence rates compared to both Region 2 (Abeline) and Region 3 (Arlington). Lastly, Region 6 (Houston) exceeded all regions based on the odds ratio and confidence intervals

**Table 2.** Adjusted Association between Individual- and Area-level Factors with HPV Vaccine Initiation among Texas Medicaid Enrollees Ages 9 to 18 Years.

Variable	OR (95% CI)
Total person-years	2 757 577
Sex	
Female	REF
Male	**0.97 (0.96, 0.98)**
Race/ethnicity	
Non-Hispanic White	REF
Non-Hispanic Black	**1.17 (1.15, 1.18)**
Non-Hispanic Asian	**1.27 (1.23, 1.31)**
Hispanic	**1.38 (1.36, 1.40)**
Other/missing	**1.07 (1.05, 1.09)**
Urbanicity of residence	
Metro	REF
Rural	**0.79 (0.76, 0.83)**
Urban	**0.86 (0.85, 0.87)**
Percent of high school degree per zip code	
<70.0	REF
70.0-80.8	**0.98 (0.97, 0.99)**
80.9-88.2	**0.94 (0.93, 0.95)**
>88.2	**0.89 (0.88, 0.90)**
Texas Public Health Region	
1 – Northwest (Lubbock)	REF
2 – North (Abilene)	**1.05 (1.01, 1.09)**
3 – North (Arlington)	**1.08 (1.05, 1.10)**
4 – Northeast (Tyler)	**1.13 (1.10, 1.16)**
5 – East Texas	**1.07 (1.04, 1.11)**
6 – Southeast (Houston)	**1.25 (1.22, 1.28)**
7 – Central (Austin)	**1.16 (1.13, 1.18)**
8 – South Central (San Antonio)	**1.12 (1.09, 1.14)**
9 – West (Odessa)	**0.88 (0.85, 0.91)**
10 – West Border (El Paso)	**1.15 (1.12, 1.18)**
11 – South Border (Harlingen)	**1.14 (1.11, 1.17)**
Cohort Year	
2019	REF
2020	**0.80 (0.79, 0.81)**
2021	**0.86 (0.85, 0.87)**

Abbreviations: CI, confidence interval; OR, odds ratio.

Urbanicity of residence is based on RUCC.

Bold signifies statistical significance.

Males were significantly less likely than females to be vaccinated for HPV, and the 2020 and 2021 cohorts were significantly less likely to be vaccinated for HPV than the 2019 cohort. All racial/ethnic groups were significantly more likely to be vaccinated for HPV compared to their White counterparts. Groups that were less likely to be vaccinated for HPV included rural and urban areas compared to metropolitan areas and zip codes with higher educational attainment.

### Regional Differences in HPV Vaccine Initiation

**Figure 1.**
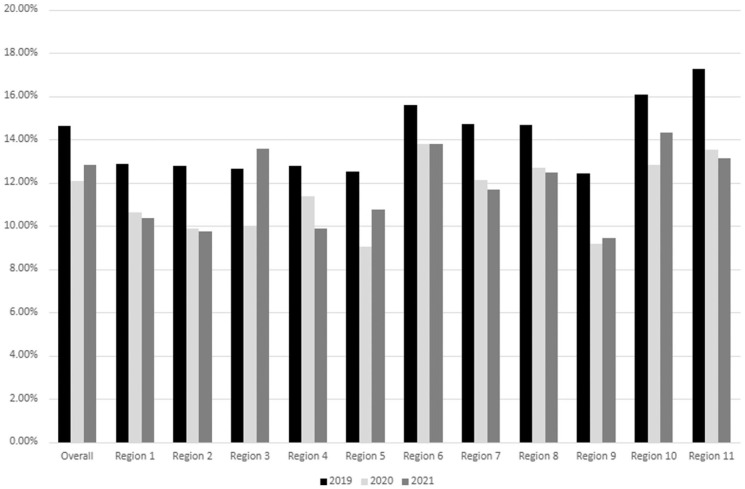
Incidence rate of HPV vaccination among Medicaid enrollees aged 9 to 18 years by Texas region. Region 1: Northwest (Lubbock); Region 2: North (Abilene); Region 3: North (Arlington); Region 4: Northeast (Tyler); Region 5: East Texas; Region 6: Southeast (Houston); Region 7: Central (Austin); Region 8: South Central (San Antonio); Region 9: West (Odessa); Region 10: West Border (El Paso); Region 11: South Border (Harlingen).

There was a statistically significant interaction between cohort year and region for HPV vaccination. Thus, the rates of HPV vaccination are presented in a figure format stratified by region and year (Figure 1).

Region 11 (South Border) had the highest HPV vaccination rate in 2019, and in 2021, the highest rate was in Region 10 (West Border). Most regions experienced a decline in HPV vaccination rates from 2019 to 2021, except for Region 3 (North).

### HPV-related CPRIT-funded Projects in Regions

Given the variation of regional HPV vaccination incidence rates over time, we aimed to understand potential drivers of the differences, such as state-funded projects for HPV vaccination through CPRIT. We limited the cohort to the years 2020 to 2021 so we could examine the association between the ratio of HPV-related CPRIT prevention programs in a year and the number of counties in a public health region in 2019 to 2020. Adjusting for sex, race/ethnicity, urbanicity, community-level educational attainment, and cohort year, those in Public Health regions with more CPRIT-funded HPV prevention projects were significantly more likely to be vaccinated for HPV (OR = 1.04, 95% CI 1.04, 1.05).

## Discussion

This paper examined the regional variations in HPV vaccine initiation incidence among an adolescent Medicaid population in Texas. Unfortunately, HPV vaccination incidence rates declined over time overall; however, not all Texas public health regions experienced the same level of decline. Moreover, regions with more HPV-related CPRIT-funded prevention grants were more likely to have higher HPV vaccination rates.

The findings from this analysis demonstrate the need to consider regional differences in HPV vaccination among Medicaid recipients in Texas. Higher incidence rates of HPV vaccination were observed in Harris County (City of Houston) and border counties, which have traditionally demonstrated elevated rates compared to the rest of Texas, according to the National Immunization Survey (NIS)-Teen data.^7^ Furthermore, Texas’s investment in HPV-related cancer prevention through CPRIT has a demonstrated association with the HPV vaccination rates among Medicaid populations. This is likely due to CPRIT’s emphasis on medically underserved populations as part of the prevention programming.^12^

Our analysis of Texas Medicaid data from 2019 to 2021 revealed a sharp, pandemic-driven decline in HPV vaccination initiation in 2020, a finding consistent with national patterns. This decline occurred in all Texas public health regions. Broader studies confirm this was a widespread issue, with U.S. claims data showing a substantial decline in adolescent vaccinations^13^ that disproportionately affected vulnerable populations and exacerbated existing disparities.^13^ The stagnation of rates into 2021 underscores the national challenge of recovery, as projections show a simple return to pre-pandemic rates is insufficient to reverse the deficit of missed opportunities.^14,15^

The results from this analysis should be considered within the context of its limitations. This cohort includes only those with Medicaid who had continuous enrollment for a 2-year period. This cohort excludes those who changed insurance or lost insurance during this time. Furthermore, since HPV vaccination is recommended at ages 9 to 11 for initiation of the series, older adolescents may have been previously vaccinated, but it is not captured in the dataset. This analysis does not measure the prevalence of HPV vaccination; rather, the outcome is an observed incidence of HPV vaccination during the study period. Lastly, while we capture the number of HPV-related CPRIT-funded projects in each region relative to the number of counties in a region, each project may vary in its priority populations and strategies.

Declines in HPV vaccination after the COVID-19 pandemic among Texas Medicaid-insured adolescents are of urgent concern if HPV-related cancers are to be prevented. More attention is needed for the regions of Texas that have persistently low rates of HPV vaccine uptake, and how strategies may need to be tailored for those areas of the state.
